# Zoledronate Inhibits Ischemia-Induced Neovascularization by Impairing the Mobilization and Function of Endothelial Progenitor Cells

**DOI:** 10.1371/journal.pone.0041065

**Published:** 2012-07-25

**Authors:** Shih-Hung Tsai, Po-Hsun Huang, Wei-Chou Chang, Hsiao-Ya Tsai, Chih-Pei Lin, Hsin-Bang Leu, Tao-Cheng Wu, Jaw-Wen Chen, Shing-Jong Lin

**Affiliations:** 1 Department of Emergency Medicine, Tri-Service General Hospital, National Defense Medical Center, Taipei, Taiwan; 2 Institute of Clinical Medicine, National Yang-Ming University, Taipei, Taiwan; 3 Division of Cardiology, Department of Internal Medicine, Taipei Veterans General Hospital, Taipei, Taiwan; 4 Cardiovascular Research Center, National Yang-Ming University, Taipei, Taiwan; 5 Department of Radiology, Tri-Service General Hospital, National Defense Medical Center, Taipei, Taiwan; 6 Division of General Laboratory, Department of Pathology and Laboratory Medicine, Taipei Veterans General Hospital, Taipei, Taiwan; 7 Institute and Department of Pharmacology, National Yang-Ming University, Taipei, Taiwan; 8 Healthcare and Management Center, Taipei Veterans General Hospital, Taipei, Taiwan; 9 Department of Medical Research and Education, Taipei Veterans General Hospital, Taipei, Taiwan; Emory University School of Medicine, United States of America

## Abstract

**Background:**

Bisphosphonates are a class of pharmacologic compounds that are commonly used to treat postmenopausal osteoporosis and malignant osteolytic processes. Studies have shown that bone marrow-derived endothelial progenitor cells (EPCs) play a significant role in postnatal neovascularization. Whether the nitrogen-containing bisphosphonate zoledronate inhibits ischemia-induced neovascularization by modulating EPC functions remains unclear.

**Methodology/Principal Findings:**

Unilateral hindlimb ischemia was surgically induced in wild-type mice after 2 weeks of treatment with vehicle or zoledronate (low-dose: 30 μg/kg; high-dose: 100 μg/kg). Doppler perfusion imaging demonstrated that the ischemic limb/normal side blood perfusion ratio was significantly lower in wild-type mice treated with low-dose zoledronate and in mice treated with high-dose zoledronate than in controls 4 weeks after ischemic surgery (control vs. low-dose vs. high-dose: 87±7% vs. *61±18% vs. **49±17%, *p<0.01, **p<0.005 compared to control). Capillary densities were also significantly lower in mice treated with low-dose zoledronate and in mice treated with high-dose zoledronate than in control mice. Flow cytometry analysis showed impaired mobilization of EPC-like cells (Sca-1^+^/Flk-1^+^) after surgical induction of ischemia in mice treated with zoledronate but normal levels of mobilization in mice treated with vehicle. In addition, ischemic tissue from mice that received zoledronate treatment exhibited significantly lower levels of the active form of MMP-9, lower levels of VEGF, and lower levels of phosphorylated eNOS and phosphorylated Akt than ischemic tissue from mice that received vehicle. Results of the *in vitro* studies showed that incubation with zoledronate inhibited the viability, migration, and tube-forming capacities of EPC.

**Conclusions/Significance:**

Zoledronate inhibited ischemia-induced neovascularization by impairing EPC mobilization and angiogenic functions. These findings suggest that administration of zoledronate should be withheld in patients with ischemic events such as acute limb ischemia.

## Introduction

Angiogenesis is necessary for wound healing and is a physiological response to tissue ischemia [1]. In recent years, our understanding of the process responsible for new vessel formation in response to tissue ischemia has changed. There is increasing evidence that neovascularization in adults is not solely the result of angiogenesis but may also involve bone marrow-derived endothelial progenitor cells (EPCs) in the process of vasculogenesis [2]. These circulating EPCs can be mobilized endogenously in response to tissue ischemia or exogenously by cytokine stimulation [3], [4]. Enhanced mobilization of EPCs augments the neovascularization of ischemic tissue and may be clinically relevant in the setting of tissue ischemia [5], [6]

Bisphosphonates (BPs) are a class of pharmacologic compounds that are commonly used to treat postmenopausal osteoporosis and malignant osteolytic processes such as multiple myeloma and complications associated with cancer metastasis to bone [7]. Their antiresorptive mechanism results in a significant reduction in skeletal-related events such as bone pain, hypercalcemic episodes, and fractures. Newly developed nitrogen-containing bisphosphonates (N-BPs), such as zoledronate, have been shown to have more potent therapeutic effects. However, protracted use of N-BPs has been demonstrated to result in the development of bisphosphonate-related osteonecrosis of the jaw (ONJ) [8]–[10]. ONJ is a rare but serious complication of N-BP treatment and has been attributed to N-BP-induced antiangiogenic effects. Recent studies have shown that N-BPs can promote osteoclast apoptosis, attenuate the activity of matrix metalloproteinases (MMPs), and inhibit farnesyl pyrophosphate synthase, a crucial enzyme in the mevalonate pathway [11]. In addition, metronomic weekly use of low-dose zoledronate has been shown to result in sustained suppression of vascular endothelial growth factor (VEGF) expression in breast cancer patients [12]. These findings suggest that zoledronate and other N-BPs play a critical role in the modulation of ischemia-induced neovascularization and the functionality of EPCs. In this study, we evaluated the possible mechanisms governing the effects of zoledronate on ischemia-induced neovascularization.

## Results

### Zoledronate inhibits blood flow recovery in the ischemic hindlimb

**Figure 1:**
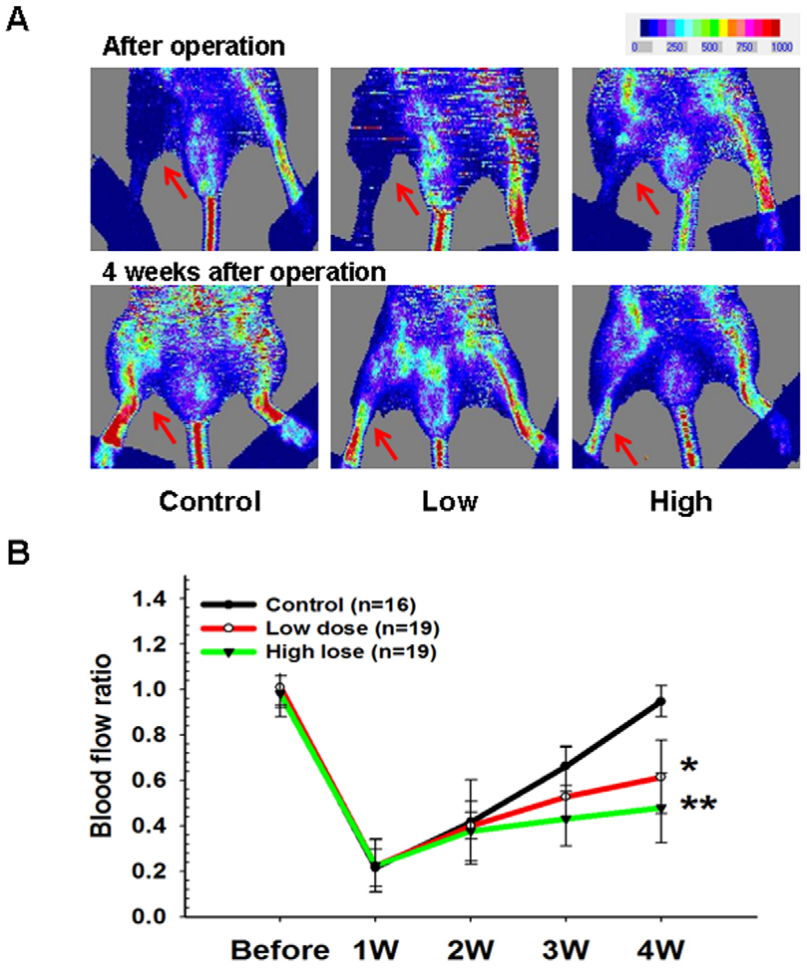
Zoledronate impaired ischemia-induced neovascularization. (A) Representative results of laser Doppler measurements before operation and 4 weeks after hindlimb ischemia surgery in controls and in low-dose and high-dose zoledronate-treated mice. Color scale illustrates blood flow variations from minimal (dark blue) to maximal (red) values. Arrows indicate ischemic (right) limb after hindlimb ischemia surgery. (B) At 28 days, compared to control group, both low-dose and high-dose zoledronate-treated groups had significantly lower recovery of blood flows measured by laser Doppler perfusion imaging. Results are means ± SEM. (*p<0.01, **p<0.001 compared to control; n = 16–19).

**Figure 2:**
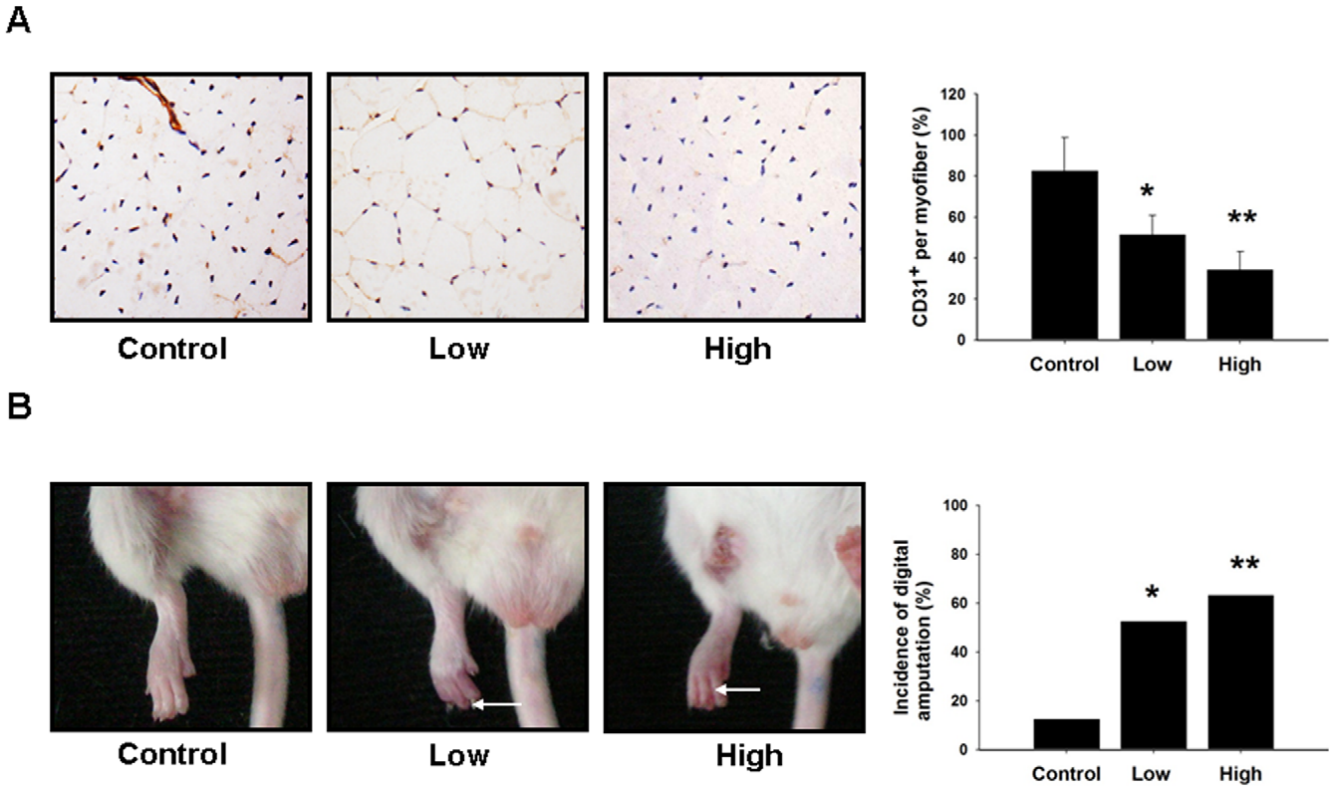
Zoledronate resulted in a decrease in new vessel formation and an increase in auto-amputation. (A) Capillary densities were significantly lower in mice treated with low-dose and high-dose zoledronate (*p<0.001, **p<0.001 compared to control). (B) Compared to the control group, the incidence of limb necrosis was also significantly higher in mice treated with low dose zoledronate and in mice treated with high dose zoledronate (*p<0.05, **p<0.005 compared to control).

To evaluate the antiangiogenic effect of zoledronate, we surgically induced unilateral hindlimb ischemia in wild-type mice. As shown in Figure 1A, laser Doppler imaging measurements revealed that blood flow in the ischemic leg of control wild-type mice recovered gradually, reaching approximately 90% of the blood flow in the untreated leg by 4 weeks (control group, n = 16), whereas blood flow recovery was significantly impaired in wild-type mice treated with low-dose zoledronate (n = 19) and in wild-type mice treated with high-dose zoledronate (n = 19) 4 weeks after ischemic surgery (control vs. low-dose vs. high-dose: 94.76±0.07% vs. *61.44±16.21% vs. **47.85±15.26%, *p<0.01, **p<0.01 compared to control; Figure 1A and B). Capillary densities were also significantly lower in mice treated with low-dose zoledronate and in mice treated with high-dose zoledronate than in control mice (control vs. low-dose vs. high-dose: capillary/myofiber ratio: 0.83±0.16 v.s. *0.51±0.10 v.s. **0.34±0.09; *p<0.01, **p<0.01, compared to control; Figure 2A and C). In addition, the incidence of limb necrosis was also significantly higher in mice treated with low-dose zoledronate and in mice treated with high-dose zoledronate than in controls (control vs. low-dose vs. high-dose: 12.5% vs. *63.2% vs. **52.6%, * p<0.05, **p<0.01, compared to control, Figure 2B and D). However, there were no significant differences in total calcium or creatinine levels among the three groups (data not shown). These findings suggest that treatment with zoledronate attenuates blood flow recovery and decreases new blood vessel formation in ischemic tissue.

### Zoledronate impairs EPC mobilization after acute ischemia

To investigate the effects of zoledronate on EPC mobilization in response to tissue ischemia, levels of Sca-1+/Flk-1+ cells in peripheral blood were determined by flow cytometry in wild-type mice and in mice treated with low-dose and high-dose zoledronate. The basal number of EPCs did not differ significantly between the wild-type control mice and mice treated with low-dose or high-dose zoledronate (Figure 2A). Consistent with previous study regarding tissue ischemia can induce EPC mobilization, EPC mobilization was enhanced after hindlimb ischemia surgery in wild-type mice (baseline vs. 2 days after operation: control, 5±4.24 vs. 24.3±10.97/10^6^MNCs, P<0.05). However, impaired mobilization of the EPCs in the peripheral blood was observed in mice treated with low-dose and high-dose zoledronate after hindlimb ischemia surgery (low-dose zolendronate, 5±1.73 vs. 14±5.20/10^6^MNCs, P = 0.17; high-dose zoledronate, 7±4.95 vs. 8.33±3.51/10^6^MNCs, P  = 0.42).

**Figure 3:**
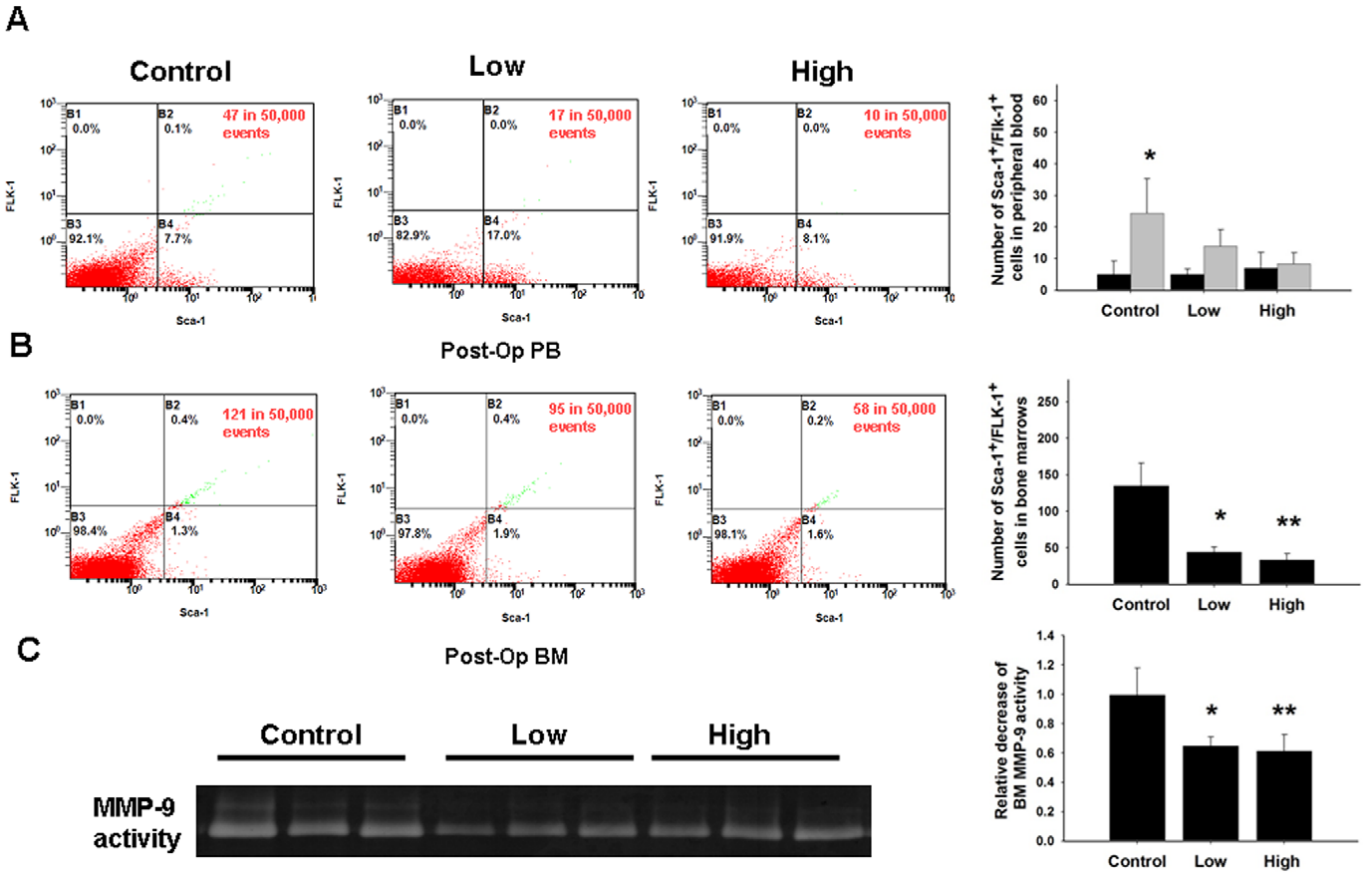
Zoledronate treatment resulted in impaired EPC mobilization and reduced bone marrow EPC number. (A) The number of Sca-1^+^/Flk-1^+^ cells in peripheral blood mononuclear cells was examined by an fluorescence-activated cell sorter. Circulating EPCs were quantified by enumerating Sca-1^+^/Flk-1^+^ cells, and the number of EPCs was determined by flow cytometry before and after hind limb ischemia surgery (24 hours) in wild-type mice and FHL2^−/−^ mice. Impaired mobilization of EPCs in peripheral blood was observed in mice treated with low-dose zoledronate and in mice treated with high-dose zoledronate after hindlimb ischemia surgery. (*P<0.05 compared to before operation; n = 6 for each group) (B) BM EPCs were determined 2 days after hindlimb ischemic surgery in study groups. (*p<0.05, **p<0.05 compared to control; n = 6 for each group). (C) Zymographic detection of MMP-9 gelatinolytic activities in BM tissues were assessed in untreated wild-type mice and in mice treated with low-dose or high-dose zoledronate (*p<0.001, **p<0.001 compared to control; n = 6 for each group).

EPCs in bone marrow were determined 2 days after hindlimb ischemic surgery. There was a significantly higher number of EPC-like cells in bone marrow in the control group than in bone marrow in the low-dose and high-dose zoledronate-treated groups (control vs. low-dose vs. high-dose group: 119.8±45 v.s. *40.2±9.4 v.s. **33.±8.6, *p<0.05, **p<0.05, compared to control; Figure 3B).

MMP-9 has been shown to play a critical role in the process of ischemia-induced EPC mobilization. We, therefore, used a zymographic assay to determined MMP-9 activity in bone marrow tissues. As shown in Figures 3C, bone marrow tissues in the control group exhibited significantly higher levels of MMP-9 activity than bone marrow tissues in the low-dose and high-dose zoledronate-treated groups (control vs. low-dose vs. high-dose zoledronate: 1±0.21 vs. *0.69±.0.03 vs. **0.67±0.10, *p = <0.01, **p = <0.01, compared to control).

### Effects of zoledronate on VEGF, eNOS, Akt, and MMP-9 activities in ischemic tissues

**Figure 4:**
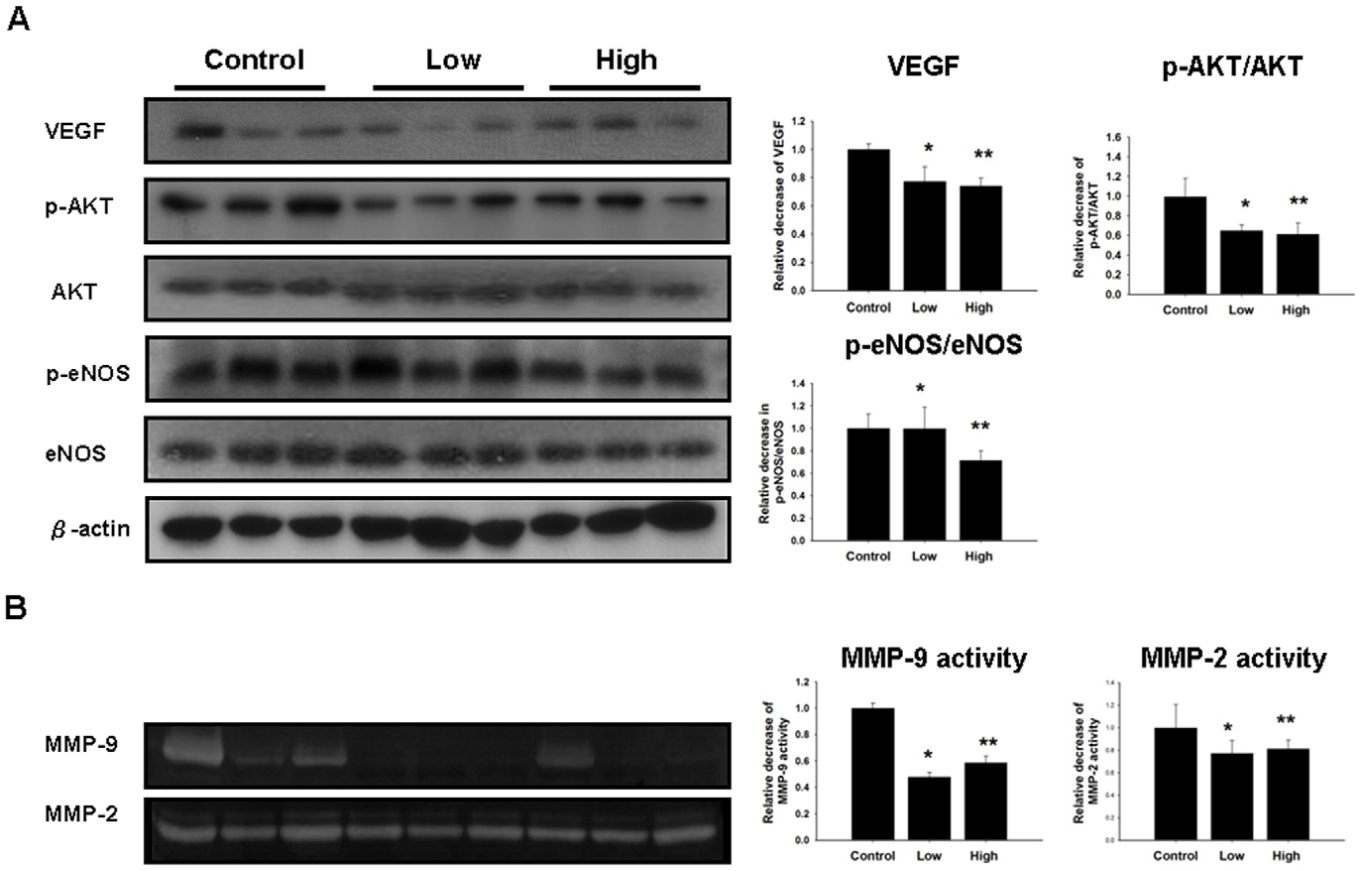
Zoledronate treatment resulted in decreased expression of VEGF and downregulation of eNOS, Akt and MMP-9 activities in ischemic tissue. (A) Zoledronate decreased the expression of VEGF (*p<0.05, **p<0.005 compared to control; n = 6 for each group). High-dose zolendronate significantly downregulated eNOS activity in ischemic muscle (*p<0.05 compared to control), and decreased Akt activity both in low-dose and high-dose regimens (*p<0.05, **p<0.05 compared to control). (B) Zoledronate inhibited MMP-9 activity in ischemic hindlimbs (*p<0.05, ** p<0.05 compared to control). However MMP-2 activity was not significantly inhibited (*p = 0.17; **p = 0.21, compared to control).

We further evaluated the effect of zoledronate on VEGF, eNOS, Akt, and MMP-9 activities in ischemic muscle of mice by Western blotting and zymography, as shown in Figure 4. Administration of zoledronate resulted in lower levels of VEGF expression (control v. low-dose vs. high-dose zoledronate: 1±0.04 vs. *0.77±.0.10 vs. **0.74±0.06, *p<0.05; **p<0.01, compared to control). Furthermore, administration of high-dose but not low-dose zoledronate significantly downregulated the activity of eNOS in ischemic hindlimbs (p-eNOS/total eNOS, control vs. low-dose vs. high-dose zoledronate: 1±0.12 vs. *1±.0.19 vs. **0.71±0.08, *p = 0.98, **p<0.05, compared to control). In addition, treatment with low-dose and treatment with high-dose zoledronate downregulated the activity of Akt (p-Akt/Akt, control vs. low-dose vs. high-dose zoledronate: p-Akt/Akt, 1±0.18 v.s. 0.64±.0.06 v.s. 0.61±0.11, *p<0.05, **p<0.05, compared to control). Zoledronate, both at low dose and at high dose, also inhibited the activity of MMP-9 (control vs. low-dose vs. high-dose zoledronate: 1±0.04 v.s. 0.48±.0.03 v.s. 0.59.±0.05, *p = 0.036, **p = 0.038, compared to control) in ischemic hindlimbs. However, MMP-2 activity was not significantly inhibited (control vs. low-dose vs. high-dose zoledronate: 1±0.2 v.s. 0.77±.0.11 v.s. 0.81.±0.07, *p = 0.17; **p = 0.21, compared to control).

### Characterization of human EPC

**Figure 5:**
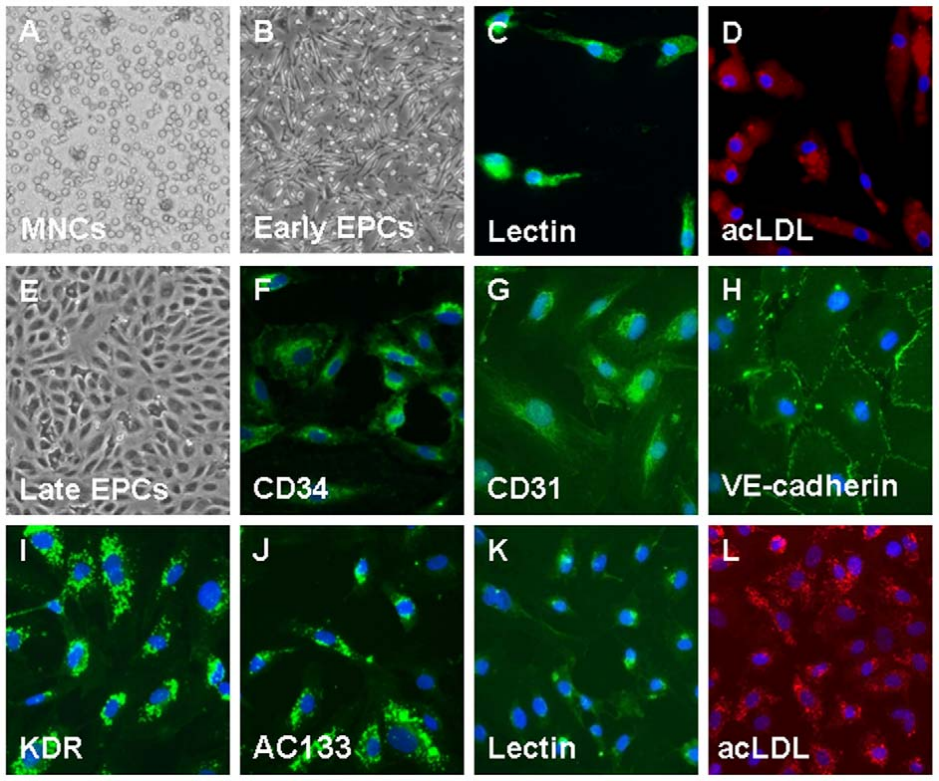
Morphology and characterization of human EPCs from peripheral blood. MNCs were plated on a fibronectin-coated culture dish on the first day (A). Four days after plating, adherent early EPCs with a spindle shape were shown (B). Three weeks after plating, endothelial colony-forming cells (ECFCs) with a cobblestone-like morphology were selected, reseeded, and grown to confluence (E). ECFCs were characterized by immunofluorescence detection of CD34 (F), CD31 (G), VE-cadherin (H), KDR(I), and AC133 (J). Early EPCs and ECFCs were shown to simultaneously bind fluorescein isothiocyanate UEA-1 (lectin, green; C, K) and endocytose DiI-acLDL (red; D, L). Cells were counterstained with DAPI for the nuclei (blue).

As shown in Figure 5, MNCs were plated on a fibronectin-coated culture dish on the first day (A). Four days after plating, adherent early EPCs with a spindle shape were shown (B). Most cells were shown to simultaneously bind fluorescein isothiocyanate UEA-1 (lectin, green, C) and endocytose DiI-acLDL after cultured for 4–7 days (red, D). Three weeks after plating, late EPCs with a cobblestone-like morphology were selected, reseeded, and grown to confluence (E) Late EPCs were characterized by immunofluorescence detection of CD34 (F), CD31 (G), VE-cadherin (H), KDR (I) and AC133 (J). Most cells were shown to simultaneously bind fluorescein isothiocyanate UEA-1 (lectin, green, K) and endocytose DiI-acLDL (red, L).

### Zoledronate inhibits the viability and enhances senescence and apoptosis of EPCs

**Figure 6:**
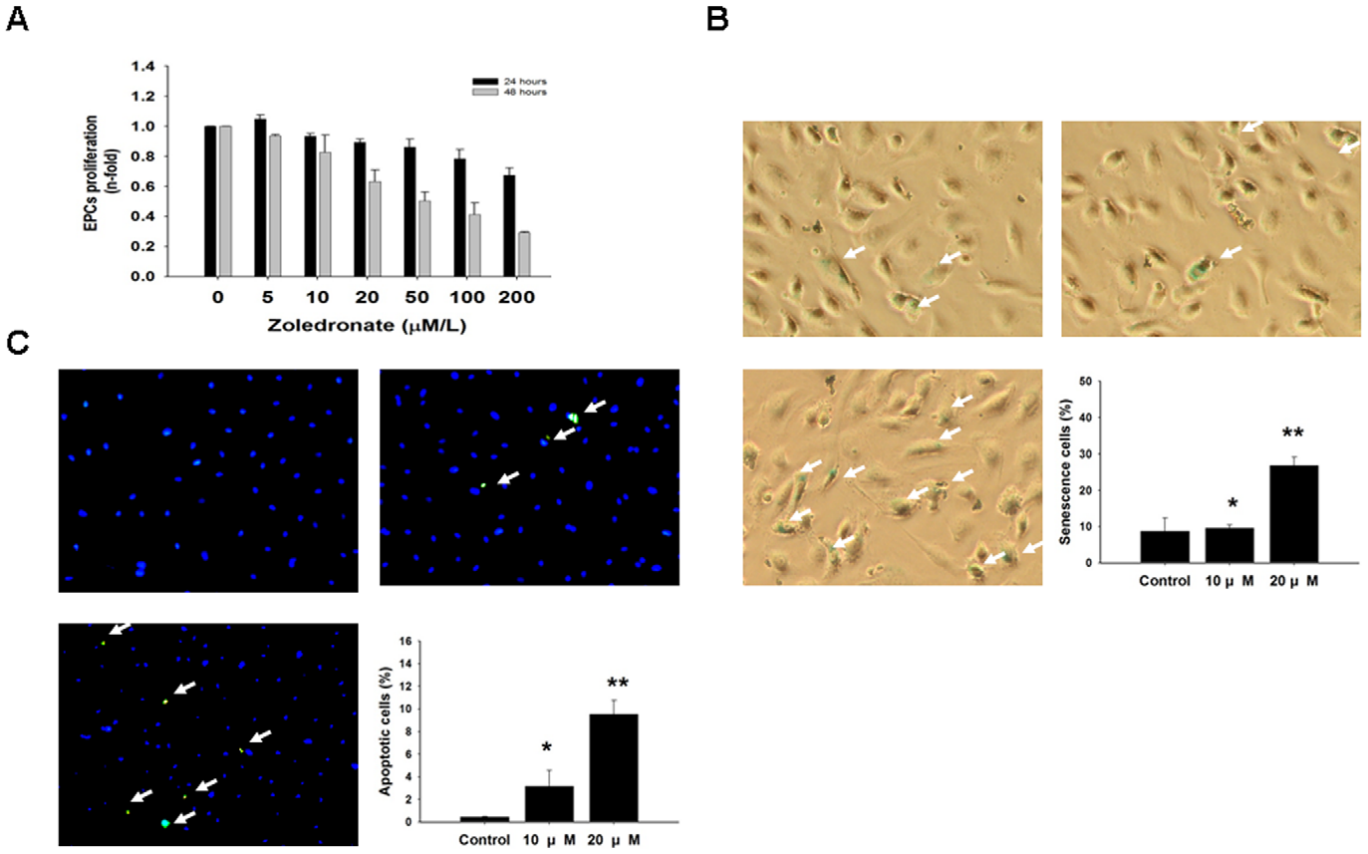
Effects of zoledronate on EPCs viability, senescence, and apoptosis. (A)\The effects of zoledronate on EPC viability were analyzed by the MTT assay. (*p<0.05 compared to control.) (B) To determine the onset of cellular aging, acidic ß-galactosidase was used as a biochemical marker for acidification, typical of EPC senescence. (*p<0.005 compared to control.) (C) Detection of apoptosis of EPCs was performed with a terminal deoxynucleotidyl transferase-mediated dUTP nick end-labeling (TUNEL) assay. (*p<0.05, **p<0.001 compared to control group**)** (n = 4 for each experiment).

As shown in Figure 6A, zoledronate inhibited the viability of EPC in a time- and dose-dependent manner. Moreover, incubation of zoledronate increased cellular senescence (control vs. 10µM vs. 20µM, 8.74±3.57 vs. *9.58±0.01 vs. **2.69±2.30%, *p = 0.72, **p< 0.01, compared to control; Figure 6B), and apoptosis (0.46±0.02 vs. *3.15±1.41 vs. **9.53±1.22%; * p<0.05 **, p<0.01, compared to control group; Figure 6C) of EPCs.

### Zoledronate suppresses the migration and tube-forming capacities of EPCs

**Figure 7:**
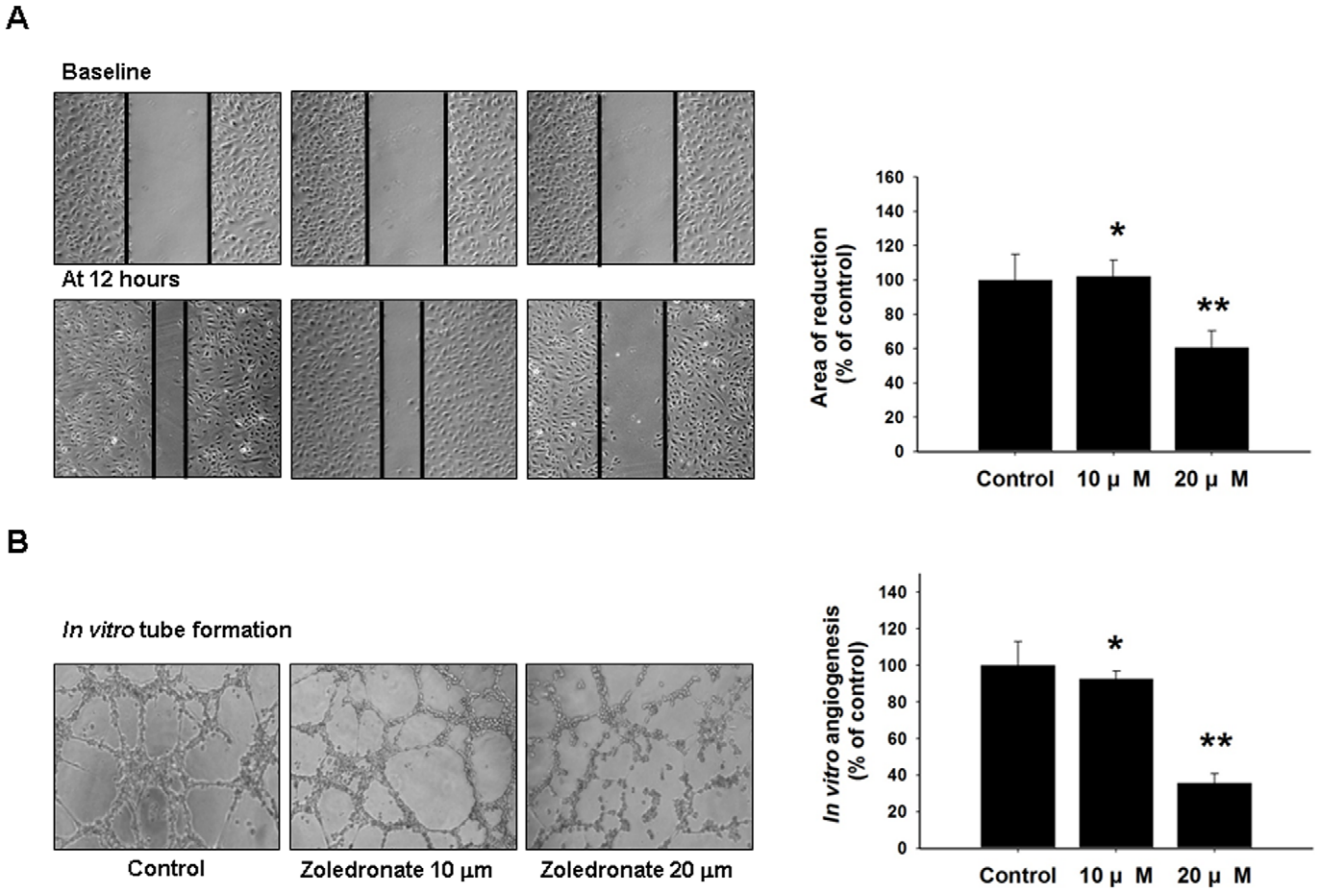
Zoledronate inhibited migration and tube formation of EPCs *in vitro.* (A) The migratory function of EPCs was evaluated by a scratch injury model. Zoledronate significantly impaired EPC migration (*p<0.05 compared to control). (B) An *in vitro* angiogenesis assay for EPCs was carried out using ECMatrix gel. Representative photos of in vitro angiogenesis are shown. Cells were stained with crystal violet, and the averages of the total area of complete tubes formed by cells were counted. (*p<0.001 compared to control).

Treatment with zoledronate resulted in significant impairment of EPC migration relative to controls (control vs. 10μM vs. 20μ M, 100±15.03 vs. *102.22±9.34 vs. **60.48±10.01%, *p =  0.84, **p<0.05, compared to control; Figure 7A). In addition, incubation with zoledronate resulted in marked suppression of EPC tube-forming capacity (100±12.85 vs. *92.66±4.14 vs. **35.68±5.09%, *p = 0.25, **p<0.001, compared to control; Figure 7B).

## Discussion

We found that administration of zoledronate at clinically relevant concentrations attenuated blood flow recovery and diminished neovascularization in wild-type mice that had undergone surgically induced hindlimb ischemia. In addition, we found that treatment with zoledronate downregulated VEGF, eNOS, Akt, and MMP-9 activities in ischemic tissues, resulted in reduced numbers EPC-like cells in bone marrow, and inhibited the mobilization of EPCs in response to acute ischemia. The in vitro studies further demonstrated that zoledronate suppressed EPC viability, EPC migration, and EPC tube-forming capacity. These findings support those presented in previous studies [13], [14], which suggested that the antiresorptive effects of zoledronate might be attributed to its antiangiogenic effect, and that this effect may be mediated by the drug's ability to downregulate the mobilization of EPCs, impair vasculogenesis, and inhibit the generation of new blood vessels in ischemic tissues.

Bisphosphonates (BPs) are a class of pharmacologic compounds that are commonly used to treat postmenopausal osteoporosis and osteolytic diseases such as multiple myeloma and complications associated with cancer metastasis to bone [7]. Newly developed N-BPs such as zoledronate have been shown to have more potent therapeutic effects. Zoledronate is very effective at suppressing osteoclastic activity and resorptive bone loss and is, therefore, widely used to treat various bone diseases, including postmenopausal osteoporosis. However, protracted use of N-BPs increases the risk of ONJ and delayed wound healing [8]–[10]. It is, therefore, necessary to examine the risk-to-benefit ratio for this drug. To the best of our knowledge, however, the effects of zoledronate on blood flow recovery and neovascularization in ischemic tissue have never been investigated.

Neovascularization at the ischemic tissue requires not solely angiogenesis but also circulating EPCs during vasculogenesis [2]. These circulating EPCs are derived from bone marrow and are mobilized in response to ischemia [3], [4]. An inadequate angiogenic response to ischemia in the myocardium or peripheral ischemic limbs of patients might result in poor collateral formation and severe organ damage. Recently, it has become clear that the role of MMPs in angiogenesis is more complex than simply degrading the extracellular matrix to facilitate invasion of endothelial cells [15]. MMP-2 and MMP-9 have been shown to play important roles in the initiation of angiogenesis [16], have been shown to be upregulated in ischemic tissue [15]–[17], and have been shown to promote the release of extracellular matrix-bound cytokines that regulate angiogenesis [18]. In the present study, treatment with zoledronate attenuated blood flow recovery and diminished new vessel formation in mice with acute hindlimb ischemia. These effects may be due to the ability of zoledronate to inhibit EPC mobilization and vasculogenesis in ischemic tissue. Furthermore, we found that treatment with zoledronate inhibited MMP-9 activity in bone marrow tissue and in ischemic muscle. This finding is consistent with that reported previously by Giraudo et al., who showed that treatment with N-BPs resulted in a reduction in MMP-9 expression in osteoclasts [19]. Likewise, N-BPs have been shown to inhibit the differentiation of osteoclasts from macrophages, inhibit the functions of matured osteoclasts, and to induce apoptosis in osteoclasts [20]–[23]. A recent study also indicated that zoledronate can suppress MMP-9 expression by infiltrating macrophages, can inhibit MMP activity by chelating zinc, and can reduce the association of VEGF with its receptor on angiogenic endothelial cells [24]. Bone-bound BPs can also suppress the growth of adjacent non-bone cells [25]. In addition, mice treated with zoledronate have been shown to have significantly lower neutrophil counts, impaired neutrophil chemotaxis, and impaired neutrophil NADPH oxidase activity [26]. Zoledronate-treated rats have also been shown to have lower levels of CD 68 cells in the marrow of long bones [27]. In addition, neutropenia has been reported in patients treated with BPs [28]–[30]. In this study, we have shown that zoledronate influences the microenvironment of bone marrow and impairs the viability and mobilization of EPCs in ischemic tissue. A large body of evidence indicates that MMP-9 is essential for ischemia-induced neovascularization because that metalloproteinase is involved in the rapid release of stem cell-active cytokines that modulate bone marrow-derived EPCs [15], [16], [18]. However, Basi *et* *al*. reported that zoledronate-treated animals showed a significant increase in MMP-9 activity [31]. The differences between the findings in our study and those in the study by Basi et al. could be due to the different animal models used the two studies. More studies are needed to clarify the role of MMP-9 expression in zoledronate treatment.

N-NPs can inhibit farnesyl pyrophosphate synthase (FPPS) in the mevalonate pathway. The mevalonate pathway is important for the production of small G-proteins, such as Ras, Rac, and Rho, that are important for intracellular structure, signaling, and transport. In vitro, N-BPs inhibit angiogenesis at low concentrations independent of FPPS; however, in vivo, higher concentrations are required to inhibit FPPS [32], [33]. Zoledronate elicits cell-cycle prolongation that seems to be associated with alterations in the levels of certain cyclins and cyclin-related regulatory proteins [34]. In vitro, zoledronate has been shown to inhibit the proliferation, migration, and adhesion of mature endothelial cells, fibroblasts, epithelial cells, vascular smooth muscle cells and oral keratinocytes [35], [36]. Consistent with previous studies, the data derived from the current study indicate that administration of clinically relevant doses of zoledronate inhibits the survival of EPCs by promoting EPC apoptosis. In addition, our findings show that zoledronate inhibits the viability, migration, and tube-forming capacities of EPC.

Minodronate, a novel third-generation nitrogen-containing bisphosphonate, was shown to inhibit VEGF-induced DNA synthesis and tube formation in endothelial cells by suppressing NADPH oxidase-mediated reactive oxygen species generation and Ras and Rho activation [37]. Although most clinicians now agree that dental extraction should be avoided in patients with myeloma who are taking BPs and that the use of BPs should be withheld for at least one month before the procedure and should not be resumed until the patient has fully recovered and the wound has fully healed [38], clinicians should be aware that N-BPs can have sustained effects on the bone microenvironment. Another interesting finding is that zoledronate inhibited eNOS and Akt phosphorylation in ischemic tissues, which implies that treatment with zolendronate might reduce NO bioavailability. Recent evidence suggests that the mobilization and differentiation of EPCs are modified by NO, and that bone marrow-expressed eNOS is essential for the mobilization of stem cells and progenitor cells in vivo [39]. In this study, we showed that zoledronate attenuated blood flow recovery and reduced ischemia-induced neovascularization by downregulating EPC mobilization and resulted in a reduction in tube formation by attenuating the activity of MMP-9, eNOS, and Akt. Clinicians need to be aware of the distinct pharmacological properties of nitrogen-containing bisphosphonates, including long-term storage of such compounds in bone and the possible effects of such a reservoir on the microenvironment of bone marrow and modulation of hematopoietic cells under stress.

### Conclusion

This study showed that zoledronate inhibited ischemia-induced neovascularization by impairing EPC mobilization. Accordingly, patients with ischemic events such as acute limb ischemia should not be administered N-BPs. Studies regarding the use of N-BPs in the management of non-cancerous pathological neovascularization with targeted delivery should be further explored.

## Materials and Methods

### Animals

FVB mice (n = 54) were purchased from the National Laboratory Animal Center, Taiwan. Zoledronate (1-hydroxy-2-imidazol-1-yl-1-phosphono-ethyl phosphonic acid, Zometa, Norvatis Pharma AG, Basel, Switzerland) was dissolved in distilled water and stored at 4°C. For long-term storage, aliquots were kept at −80°C. The compound was filter-sterilized before use. In this study, we tested the effects of two different doses. Mice in the low-dose group were administered 30 μg/kg via subcutaneous injection since one week prior to surgical induction of ischemia and on a weekly basis. This dosing schedule was chosen because the drug level is similar to that achieved with a clinical dosing regimen of 4 mg zoledronate for the treatment of bone metastases [24]. Mice in the high-dose group received 100 μg/kg via subcutaneous injection since one week prior to surgical induction of ischemia and on a weekly basis. All experimental protocols and procedures were approved by the institutional animal care committee of the National Defense Medical Center (Taipei, Taiwan) and complied with the Care and Use of Laboratory Animals published by the US National Institutes of Health (NIH Publication No. 85-23, revised 1996).

### Mouse model of hindlimb ischemia

In the present study, we used 8-week-old male wild-type mice (C57BL/6J background). Hindlimb ischemia was induced surgically as previously described [18], [40]. Mice were anaesthetized by an intraperitoneal injection of ketamine (100 mg/kg) and xylazine (10 mg/kg). The depth of anesthesia was checked by ensuring that noxious pinch stimulation (blunt forceps) of the hindpaw, the forepaw, and the ear did not evoke motor reflexes. The proximal and distal portions of the femoral artery were ligated. Hindlimb blood perfusion was measured with a laser Doppler perfusion imaging system (Moor Instruments Limited, Devon, UK) immediately after surgery and then on a weekly basis. To avoid the influence of ambient light and temperature, the results were expressed as the ratio of perfusion in the right (ischemic) limb versus perfusion in the left (non-ischemic) limb. Limb necrosis indicates the case that foot or toe is impaired but knee is intact, while limb loss (autoamputation) means loss of limb above the knee.

### Measurement of capillary density in the ischemic limb

At 4 weeks after surgery, the mice were euthanized via intravenous ketamine injection. The limbs were fixed overnight in methanol. The femora were carefully removed and the ischemic thigh muscles were embedded in paraffin. Sections (5 μm) were deparaffinized and incubated with rat monoclonal antibody against murine CD31 (BD PharMingen, San Diego, CA). Antibody distribution was visualized with the use of the avidin-biotin-complex technique and high-sensitivity diaminobenzidine (DAB+) chromogenic substrate system (Dako Denmark), followed by counterstaining with hematoxylin. Capillaries were identified based on the presence of morphologic characteristics as well as positive staining for CD31. Visible capillaries were counted in ten randomized selected fields from each tissue preparation. Capillary density was expressed as the number of capillaries per muscle bundle.

### Measurement of MMP activities

Gelatin zymography was used to determine the gelatinolytic activities of MMP-2 and MMP-9 in bone marrow (BM) irrigations, homogenates of muscle tissues, and in conditioned medium as previously described [14]. Briefly, equivalent amounts of sample were electrophoresed under non-reducing conditions on 7.5% SDS-polyacrylamide gels containing 0.1 mg/ml gelatin as substrate. The gels were washed in a buffer containing 2.5% Triton X-100 for one hour to remove SDS and then incubated with a substrate buffer at 37°C for 18 hours. MMP activities were quantified by denstometery scanning. Data obtained from densitometric analysis were expressed as fold-change in activity relative to controls.

### Western blotting analysis of VEGF expression, total nitric oxide synthase (eNOS) and Akt expression, and expression levels of phosphorylated eNOS and phosphorylated Akt

Protein lysates were subjected to SDS-PAGE followed by electroblotting onto a PVDF membrane. Membranes were probed with monoclonal antibodies against total eNOS and phosphorylated eNOS (p-eNOS), total AKT and phoshphorylated AKT (p-AKT), VEGF, and β-actin. Bands were visualized by chemiluminescence detection reagents. Densitometric analysis was conducted with ImageQuant (Promega) software, and data were expressed as fold-change relative to controls.

### Measurement of blood components

At the end of the study, mice were anaesthetized by an intraperitoneal injection of ketamine (100 mg/kg) and xylazine (10 mg/kg). Blood samples were obtained by left ventricular puncture and placed in tubes containing EDTA (0.5 M). Plasma was obtained by centrifugation (5000 rpm) at 4°C for 10 minutes. Plasma levels of total calcium and creatinine were determined by atomic absorption spectrophotometry (SPOTCHEM II, Arkray Kyoto, Japan).

### EPC mobilization

To evaluate the effects of zoledronate on EPC mobilization, we simultaneously collected fresh peripheral blood (PB) and BM irrigations 2 days after hindlimb ischemia. We used fluorescein isothiocynate (FITC) anti-mouse Sca-1 (ebioscience, San Diego, CA, USA) and phycoerythrin (PE) anti-mouse Flk-1 (VEGFR-2, ebioscience) antibodies. The numbers of Sca-1^+^/Flk-1^+^ cells in the BM and PB mononuclear cells (MNCs) were examined by a fluorescence-activated cell sorter (FACS caliber; Becton Dickinson, San Jose, CA, USA). Circulating EPCs were considered to be from the mononuclear cell population and were gated with double positive for Sca-1 and Flk-1. The PB/BM ratio of EPCs was also determined.

### Human EPC isolation and cultivation

Peripheral blood samples (20 mL) were obtained from healthy young volunteers and total mononuclear cells (MNCs) were isolated by density gradient centrifugation with Histopaque 1077 (1.077 g/mL; Sigma, St. Louis, MO, USA). Briefly, 5×10^6^ MNCs were plated onto fibronectin-coated 6-well plates containing 2 mL of endothelial growth medium (EGM-2 MV Cambrex; East Rutherford, NJ, USA), hydrocortisone, R^3^-insulin-like growth factor 1, human endothelial growth factor, VEGF, human fibroblast growth factor, gentamicin, amphotericin B, vitamin C, and 20% fetal bovine serum. After 4 days, the medium was changed and non-adherent cells were removed. Adherent MNCs appeared elongated with a spindle shape. A number of early EPCs were allowed to grow into colonies of endothelial colony-forming cells (ECFCs), which emerged 2–4 weeks after the start of the MNC culture. The ECFCs exhibited a cobblestone morphology and a monolayer growth pattern typical of mature endothelial cells at confluence. ECFCs were collected and used for all assays in this study. All participants gave their written informed consent. The study was approved by the Institutional Review Board of the Taipei Veterans General Hospital, Taiwan and the protocols of this study were consistent with ethical guidelines provided in the 1975 Helsinki Declaration.

### EPC Characterization

ECFCs were characterized as adherent cells that were double positive for acetylated low-density lipoprotein uptake and lectin binding by direct fluorescent staining as previously described [7], [16]. Briefly, adherent cells were first incubated with 2.4 μg/ml 1,1′-dioctadecyl-3,3,3′,3′-tetramethylindocarbocyanine perchlorate-acetylated low-density lipoprotein (DiI-acLDL; Molecular Probe) for 1 hour and then fixed in 2% paraformaldehyde and counterstained with 10 μg/ml FITC-labeled ulex europaeus agglutinin-1 (UEA-1) lectin (Sigma). Late EPCs were also characterized by immunofluorescence staining for the expression of CD34, AC133, Von Willebrand factor (vWF), VE-cadherin, platelet/endothelial cell adhesion molecule-1 (PECAM-1) (CD-31), and eNOS (Santa Cruz). The fluorescent images were recorded using a laser scanning confocal microscope.

### MTT assay

The cytotoxic effects of zolendronate on cultured EPCs were tested using the MTT assay at zolendronate concentrations of 5, 10, 20, 50, 100, and 200 μM. The proliferation of EPCs was determined by direct staining of 6 random high-power microscopic fields (×100) and by 3-(4,5-dimethylthiazol-2-yl)-2,5,diphenyltetrazolium bromide (MTT) assay, respectively [14]. In brief, after 24, 36, and 48 hours of culture, cells were treated with 10 μL of MTT (5 g/L, Sigma) and were incubated for another 6 hours. The supernatant was discarded and the cell preparations were shaken with 300 μL dimethyl sulfoxide solution for 10 minutes. The OD values were measured at 570/690 nm.

### Scratch injury model

The migratory function of EPCs was evaluated by a scratch injury model. EPCs were seeded onto 6-well cell culture plates. Once at confluence, EPCs were incubated for 48 hours with 10 μM or 20 μM zoledronate. The monolayer was wounded by scraping a line across the well using a sterile pipette tip. After injury, the monolayer was gently washed with PBS and the medium was replaced with medium containing 10% FBS. EPC sprouting from the edge of the injured monolayer was examined and photographed before and 12 and 24 hours after scratching. Migration activity of EPCs was determined by measuring the width of the wound. Data were quantified as the percent of control.

### Tube formation assay

An EPC tube formation assay was performed using the In Vitro Angiogenesis Assay Kit (Chemicon) [14]. ECMatrix gel solution was thawed overnight at 4°C, mixed with ECMatrix diluent buffer, and placed in a 96-well plate for 1 h at 37°C to allow the matrix solution to solidify. EPCs were harvested with trypsin/EDTA, as described above, and 1×10^4^ EPCs were placed onto a matrix with EGM-2 MV medium and incubated at 37°C for 16 h. Tubule formation was inspected with an inverted light microscope (100×). Tubule formation was defined as a structure exhibiting a length 4 times its width. Five independent fields were assessed in each well, and the average number of tubules/200× field was determined.

### EPC senescence assay

Cellular aging was determined with a Senescence Cell Staining kit according to the manufacturer's instructions (Sigma) [14], [15]. After being washed with phosphate-buffered saline (PBS), cultured EPCs were fixed for 6 minutes in 2% formaldehyde and 0.2% glutaraldehyde in PBS and then incubated for 12 h at 37°C in the absence of CO_2_ with fresh X-gal staining solution (1 mg/ml, 5 mmol/l potassium ferrocyanide, 5 mmol/l potassium ferricyanide, and 2 mmol/l MgCl2; pH 6). After staining, the blue-stained cells and total cells were counted and the percentage of β-galactosidase-positive cells was calculated.

### TUNEL assay

Detection of apoptosis of EPCs was performed with a terminal deoxynucleotidyl transferase-mediated dUTP nick end-labeling (TUNEL) assay (Fluorescein Direct in situ apoptosis detection kit, Chemicon, USA) according to the manufacturer's protocol [41]. Briefly, the EPCs were carefully washed twice with PBS and fixed with freshly prepared 4% paraformaldehyde for 1 h at room temperature, followed by incubation with a blocking solution (3% H_2_O_2_ in methanol) for 30 min. The EPCs were permeabilized with a freshly prepared solution containing 0.5% Triton X-100 in 0.1% sodium citrate for 1 h at 37°C. The TUNEL reaction mixture (50 μL) was then added to the samples and incubation was allowed to continue for another 1 h at 37°C in a humidified atmosphere in the dark. Later, 50 μL of converter POD was added to the samples, followed by 30 min incubation at 37°C. The chromogenic reaction was carried out with 3,3′- diaminobenzidine and terminated by the addition of tap water. The EPCs were then counterstained with DAPI and analyzed. Apoptosis was determined as the percentage of positive cells per 1,000 DAPI-stained nuclei, and EPCs were visualized under a fluorescence microscope (Nikon Eclipse 50i) at a magnification of 100×.

### Statistical analysis

All continuous variables are presented as mean ± standard error of the mean (SEM). Comparisons between groups were analyzed by analysis of variance. Statistical significance was defined as a p value less than 0.05. Analyses were performed using the statistical package SPSS for Windows (Version 16.0; SPSS, Inc; Chicago, IL, U.S.A.).
